# Synthesis of Brominated 2-Phenitidine Derivatives as Valuable Inhibitors of Cholinesterases for the Treatment of Alzheimer’s Disease

**Published:** 2014

**Authors:** Muhammad Athar Abbasi, Amna Saeed, Aziz-ur-Rehman, Khalid Mohmmed Khan, Muhammad Ashraf, Syeda Abida Ejaz

**Affiliations:** a *Department of Chemistry, Government College University, Lahore-54000, Pakistan.*; b *HEJ Research Institute of Chemistry, International Center for Chemical and Biological Sciences, University of Karachi, Karachi-75270, Pakistan. *; c *Department of Biochemistry and Biotechnology. *; d *Department of Pharmacy, The Islamia University of Bahawalpur, Bahawalpur-63100, Pakistan.*

**Keywords:** 2-phenitidine, Sulfonamide, Bromination, Acetylcholinesterase, Bytyrylcholinesterase, Lipoxygenase, ^1^H-NMR

## Abstract

The present study reports the synthesis of a series *N*-substituted derivatives of brominated 2-phenitidine. First, the reaction of 2-phenitidine (1) with benzenesulfonyl chloride (2) in aqueous media yielded *N*-(2-ethoxyphenyl) benzenesulfonamide (3), which was then subjected to bromination with bromine in the presence of glacial acetic acid to give *N*-(4,5-dibromo-2-ethoxyphenyl) benzenesulfonamide (4). Secondly, the product (4) on further treatment with alkyl/aryl halides (5a-l) in the presence of lithium hydride (LiH) produced twelve new derivatives of *N*-substituted sulfonamides (6a-l). These were characterized by ^1^H-NMR spectrum and screened against acetylcholinesterase (AChE), butyrylcholinesterase (BChE) and lipoxygenase (LOX) and were found to be valuable inhibitors of butyrylcholinesterase (BChE) and acetylcholinesterase (AChE). Few of them were also active against LOX.

## Introduction

The molecules containing functional group -SO_2_NH- called sulfonamides are considered as pharmacologically significant compounds. This group is found to be present in a variety of biologically active molecules responsible for antimicrobial activity (1-2). Many of the chemotherapeutic sulfonamide derivatives are widely used as antibacterial and antiviral agents (3,4). The mechanism of action of sulfonamide involves incorporation of 4-amino benzoic acid in folic acid pathway, as it blocks the foliate synthetase enzyme. In this respect, it offers hindrance to folic acid synthesis in bacteria, which consequently creates hurdle in production of purines (1,5). Sulfonamides have shown multiple applications in biological systems such as, anticancer, anticonvulsant, antidiuretics, anti-inflammatory, hypoglycemic, insulin releasing, carbonic anhydrase inhibitors and HIV protease inhibitors (1,4,6-8). Structurally modified sulfonamides were found potential inhibitors of histone deacetylase (HDAC) cease tumor cell growth *in-vivo* in animal cultures (9). The prominent mechanism for sulfonamide function as anticancer agents includes cell cycle perturbation in G1 phase; carbonic anhydrase and angiogenesis inhibition are known (10). It has been reported that carbonic anhydrase enzyme is causative agent of several physiological and pathological diseases including epilepsy, cancer, neuromuscular disorder, obstructive pulmonary infection, and osteoporosis (11,12). Sulfonamide derivatives have found their inhibitory activity of carbonic anhydrase (CA) by coordinating with Zn^+2^ metal ion of enzyme through its SO_2_NH^- ^anion leading to reduction of HCO_3_^- ^ion synthesis in transition state necessarily required for CA function (5,10). 

Cholinesterases are enzymes that share extensive sequence homology and distinct substrate specificity and inhibitor sensitivity. Cholinesterases are potential target for the symptomatic treatment of Alzheimer’s disease and related dementias. Acetylcholinesterase (AChE, EC 3.1.1.7) and butyrylcholinesterase (BChE, EC 3.1.1.8) consist of a family of enzymes which include serine hydrolases. The different specificities for substrates and inhibitors for these enzymes are due to the differences in amino acid residues of the active sites of AChE and BChE. The enzyme system is responsible for the termination of acetylcholine at cholinergic synapses. These are key components of cholinergic brain synapses and neuromuscular junctions. The major function of AChE and BChE is to catalyze the hydrolysis of the neurotransmitter acetylcholine and termination of the nerve impulse in cholinergic synapses (13-14). It has been found that butyrylcholinesterase (BChE, E.C 3.1.1.8) inhibition is an effective tool for the treatment of AD and related dementias. BChE is found in significantly higher quantities in Alzheimer’s plaques than in plaques of normal age-related non-demented brains. BChE is produced in the liver and enriches blood circulation. In addition, it is also present in adipose tissue, intestine, smooth muscle cells, white matter of the brain and many other tissues (15-17).

Hence, the search for new cholinesterase inhibitors is considered an important and ongoing strategy to introduce new drug candidates for the treatment of Alzheimer’s disease and other related diseases (18). These findings prompted us to explore the synthesis of different *N*-substituted sulfonamides derived from brominated 2-phenitidine and subsequent their biological screening with the aim of searching valuable cholinestrases inhibitors. 

## Experimental

*Chemistry*

TLC was performed on pre-coated silica gel G-25-UV254 plates. Detection was carried out at 254 nm, and by ceric sulphate reagent. Purity was checked on TLC with different solvent systems using ethyl acetate and *n*-hexane giving single spot. The IR spectra were recorded in KBr on a Jasco-320-A spectrophotometer (wave number in cm^-1^). ^1^H NMR spectra were recorded in CDCl_3_ on a Bruker spectrometers operating at 400 MHz. Chemical shifts are given in ppm. The assignment of chemical shifts to various protons in the synthesized molecules was also helped out with ACD ^1^H-NMR soft ware. Mass spectra (EIMS) were measured on Finnigan MAT-112 instrument. EI-MS were recorded on a JMS-HX-110 spectrometer, with a data system. The melting points were recorded on a Griffin and George melting point apparatus by open capillary tube and were uncorrected.

*Procedure for the synthesis of sulfonamide in aqueous medium*

The nucleophilic substitution reaction of amine with benzenesulfonyl chloride was carried out in the following manner: an equimolar mixture of benzenesulfonyl chloride (10.0 m mol; 1.27 mL) and 2-phenitidine (10.0 mmol; 1.43 mL) was suspended in 25 mL water. The pH of the suspension was adjusted and was maintained at 9.0 by adding aqueous solution of a base at room temperature. The reaction solution was stirred and monitored with TLC and it took two hours for the completion of reaction. Then concentrated HCl was added gradually to adjust the pH to 2.0. The precipitates were collected by filtration, washed with distilled water and dried to afford the title compound 3. The product was dissolved in methanol and re-crystallized by slow evaporation of the solvent, to generate colorless bead like crystals of *N-(*2-ethoxyphenyl) benzenesulfonamide. Yield 89 %; m.p. 88 ^°^C. 

*Bromination of N-(2-ethoxyphenyl)benzenesulfonamide *(3)

2 g of compound 3 were dissolved in 10 mL of glacial acetic acid. The bromine liquid (2 mL) was added gradually in the reaction mixture for the bromination of 3**. **The reaction mixture was stirred at room temperature for at least 2 hours and the completion of reaction was monitored by TLC. The product was filtered, washed with distilled water and dried to afford dark brown precipitates of *N-*(4,5-dibromo-2-ethoxyphenyl)benzenesulfonamide (4). Yield 98%, m.p. 145-146 ^°^C.

*General procedure for the synthesis of N-alkyl substituted sulfonamides in DMF*

The calculated amount of 4 (0.1 mmol) was taken in a round bottomed flask (50 mL), then dimethyl formamide DMF (10.0 mL) was added to dissolve it followed by the addition of lithium hydride (0.1 mmol) to the mixture. The mixture was stirred for 30 minutes at room temperature and then slowly added the alkyl halide/aryl halide to the mixture and the solution was further stirred for three hours. The progress of reaction was monitored via TLC till single spot. The product was precipitated by adding water. It was filtered, washed with distilled water and crystallized from aqueous methanol. 

*Structural Characterization*

*N-*(4,5-dibromo-2-ethoxyphenyl)benzenesulfonamide (4)

Dark brown amorphous powder, Yield 98%, m.p. 145-146 ^o^C, C_14_H_13_Br_2_NO_3_S, Mol.mass-435; ^1^H-NMR (500 MHz, CDCl_3_): δ 7.77 (s, 1H, H-6), 7.74 (dd, *J = *1.5, 8.5 Hz, 2H, H-2', H-6ʹ), 7.53 (br t, *J = *7.0 Hz, 1H, H-4ʹ), 7.43 (br t, *J *= 8.0 Hz, 2H, H-3ʹ, H- 5ʹ), 6.91 (s, ^1^H, H-3), 3.78 (q, *J = *7.0 Hz, 2H, CH_2_-1ʹʹ), 1.24 (t, *J *= 7.0 Hz , 3H, CH_3_-2'').

*N-Ethyl-N-(4,5-dibromo-2-ethoxyphenyl)benzenesulfonamide *(6a)

Brown amorphous powder, Yield 90%, m.p. 120-122 ^o^C, C_16_H_17_Br_2_NO_3_S, Mol. Mass-463; ^1^H-NMR (500 MHz, CDCl_3_): δ 7.67 (br d, *J* =7.5 Hz, 2H, H-2ʹ, H, - 6ʹ), 7.53 (br t, *J *= 7.5 Hz, ^1^H, H-4ʹ), 7.52 (s, ^1^H, H-6), 7.43 (br t, *J *= 7.5 Hz, 2H, H-3ʹ, H- 5ʹ), 7.0 (s, ^1^H, H-3), 3.62 (br q, 4H, CH_2_-1ʹʹ and CH_2_-1ʹʹʹ, merged),1.04 (t, *J* = 7.0 Hz, 3H, CH_3_-2ʹʹ), 0.94 (t, *J* = 7.0 Hz, 3H, CH_3_-2ʹʹʹ).

*N-Isopropyl -N-(4,5-dibromo-2-ethoxyphenyl)benzenesulfonamide *(6b)

Tea pink amorphous powder, Yield 84%, m.p. 128-130 ^o^C, C_17_H_19_Br_2_NO_3_S,Mol. Mass-477; ^1^H-NMR (500 MHz, CDCl_3_): δ 7.81 (br d*, J *= 7.5 Hz, 2H, H-2ʹ, H-6ʹ), 7.54 (br t, * J *= 7.0 Hz, 1H, H-4ʹ), 7.46 (br t,* J *= 7.0 Hz, 2H, H-3ʹ, H-5ʹ), 7.31 (s, 1H, H-6), 7.12 (s, ^1^H, H-3), 4.31 (septet, * J *= 6.5 Hz, ^1^H, CH-1ʹʹʹ), 3.87 (q, *J *= 7.5 Hz, 2H, CH_2_-1ʹʹ), 1.22 (t,* J *= 7.0 Hz, 3H, CH_3_-2ʹʹ), 1.06 (d, *J = *7.0 Hz, 6H, CH_3_-2ʹʹʹ, CH_3_-3ʹʹʹ).

*N-Allyl -N-(4,5-dibromo-2-ethoxyphenyl)benzenesulfonamide *(6c)

Buff amorphous powder, Yield 80%, m.p. 110-112 ^°^C,C_17_H_17_Br_2_NO_3_S,Mol. Mass-475; ^1^H-NMR (500 MHz, CDCl_3_): δ 7.66 (br d,* J *= 7.5 Hz, 2H, H-2ʹ, H-6ʹ), 7.54 (br t,* J *= 7.5 Hz, 1H, H-4ʹ), 7.51 (s, ^1^H, H-6), 7.44 (br t, *J *= 8.0 Hz, 2H, H-3ʹ, H-5ʹ), 6.97 (s, 1H, H-3), 5.74 (m, 1H, CH-2ʹʹʹ), 5.05 (dd,* J* = 1.5, 17.0 Hz, 1H, H_b-_3ʹʹʹ), 5.01 (dd*, J* = 1.5, 10.0 Hz, ^1^H, H_a_-3ʹʹʹ), 4.15 (br s, 2H, CH_2_-1ʹʹʹ), 3.60 (q, 2H, CH_2_-1ʹʹ, merged), 0.95 (t,* J* = 7.5 Hz, 3H, CH_3_-2ʹʹ).

*N-(2-Bromoethyl-N-(4,5-dibromo-2-ethoxyphenyl)benzenesulfonamide *(6d)

Brown amorphous powder, Yield 80%, m.p. 112-114 ^°^C, C_16_H_16_Br_3_NO_3_S, Mol. Mass-542; ^1^H-NMR (500 MHz, CDCl_3_): δ 7.64 (d, *J *= 7.5 Hz, 2H, H-2ʹ, H-6ʹ), 7.63 (s, ^1^H, H-6, merged), 7.56 (br t*, J* = 7.5 Hz, ^1^H, H-4ʹ), 7.44 (br t, *J *= 8.0 Hz, 2H, H-3ʹ, H-5ʹ), 7.0 (s, 1H, H-3), 3.83 (br s, 2H, CH_2_-1ʹʹʹ), 3.58 (q, 2H, CH_2_-1ʹʹ merged), 3.43 (t, *J *= 7.5 Hz, 2H, CH_2_-2ʹʹʹ), 0.92 (t, *J* = 7.0 Hz, 3H, CH_3_-2ʹʹ).

*N-Benzyl-N-(4,5-dibromo-2-ethoxyphenyl)benzenesulfonamide *(6e)

Cream brown amorphous powder, Yield 96%, m.p. 174-176 ^°^C, C_21_H_19_Br_2_NO_3_S, Mol. Mass-525; ^1^H-NMR (500 MHz, CDCl_3_): δ 7.70 (br d,* J* = 8.5 Hz, 2H, H-2ʹ, H-6ʹ), 7.56 (br t,* J* = 7.0 Hz, 1H, H-4ʹ), 7.46 (br t,* J* = 7.5 Hz, 2H, H-3ʹ,H-5ʹ), 7.32 (s, ^1^H, H-6), 7.23 (br t,* J* = 7.0 Hz, 2H, H-3ʹʹʹ, H-5ʹʹʹ, overlapped), 7.21 (br t,* J* = 8.5 Hz, 1H, H-4ʹʹʹ, overlapped), 7.17 (dd, *J* = 1.5, 7.5 Hz, 2H, H-2ʹʹʹ, H-6ʹʹʹ), 6.93 (s, ^1^H, H-3), 4.72 (br s, 2H, CH_2_-7ʹʹʹ), 3.59 (q, 2H, *J* = 7.0 Hz, CH_2_-1ʹʹ), 0.99 (t,* J* = 7.0 Hz, 3H, CH_3_-2ʹʹ). 

*N-Phenylethyl-N-(4,5-dibromo-2-ethoxyphenyl)benzenesulfonamide *(6f)

Brownish moron powder, Yield 89%, m.p. 108-110 ^°^C,C_22_H_21_Br_2_NO_3_S,Mol. Mass-539; ^1^H-NMR (500 MHz, CDCl_3_): δ 7.63 (br d, *J* = 7.5 Hz, 2H, H-2ʹ, H-6ʹ), 7.52 (br t*, J* = 7.5 Hz, 1H, H-4ʹ), 7.41 (br t, *J* = 7.5 Hz, 2H, H-3ʹ, H-5ʹ), 7.39 (s, ^1^H, H-6), 7.24 (br t, *J *= 7.5 Hz, 2H, H-3ʹʹʹ,H-5ʹʹʹ), 7.18 (br t,* J* = 7.0 Hz, 1H, H-4ʹʹʹ), 7.08 (br d, *J *= 7.5 Hz, 2H, H-2ʹʹʹ, H-6ʹʹʹ), 6.97 (s, 1H, H-3), 3.63 (m, 2H, CH_2_-8ʹʹʹ, Overlapped), 3.58 (m, 2H, CH_2_-1ʹʹ, Overlapped), 2.78 (t, *J* = 7.5 Hz, 2H, CH_2_-7ʹʹʹ), 0.90 (t, *J* = 7.0 Hz, 3H, CH_3_-2ʹʹ).

*N-Phenylpropyl-N-(4,5-dibromo-2-ethoxyphenyl)benzenesulfonamide *(6g)

Black gummy solid, Yield 75%,C_23_H_23_Br_2_NO_3_S,Mol. Mass-553; ^1^H-NMR (500 MHz, CDCl_3_): δ 7.62 (dd, *J* = 1.5, 8.5 Hz, 2H, H-2ʹ, H-6ʹ), 7.54 (br t,* J* = 8.0 Hz, 1H, H-4ʹ), 7.51 (s, ^1^H, H-6), 7.44 (br t,* J* = 8.0 Hz, 2H, H-3ʹ, H-5ʹ), 7.28 (br t, *J* = 7.5 Hz, 2H, H-3ʹʹʹ, H-5ʹʹʹ), 7.14 (br t, *J *= 7.5 Hz, ^1^H, H-4ʹʹʹ), 7.07 (br d, *J *= 7.0 Hz, 2H, H-2ʹʹʹ, H-6ʹʹʹ), 6.98 (s, ^1^H, H-3), 3.78 (q, *J *= 7.0 Hz, 2H, CH_2_-1ʹʹ), 3.64 (br t, J = 7.0 Hz, 2H,CH_2_-9ʹʹʹ), 2.61 (t, *J* = 7.5 Hz, 2H, CH_2_-7ʹʹʹ), 1.70 (quintet, J = 7.0 Hz, 2H, CH_2_-8ʹʹʹ), 1.24 (t, *J* = 7.0 Hz, 3H, CH_3_-2ʹʹ). 

*N-(4-Bromobenzyl-N-(4,5-dibromo-2-ethoxyphenyl)benzenesulfonamide *(6h)

Brown amorphous powder, Yield 80%, m.p. 162-164 ^°^C,C_21_H_18_Br_3_NO_3_S,Mol. Mass-604; ^1^H-NMR (500 MHz, CDCl_3_): δ 7.68 (br d, *J *= 7.5 Hz, 2H, H-2ʹ, H-6ʹ), 7.57 (br t, *J *= 7.0 Hz, 1H, H-4ʹ), 7.46 (br t, *J *= 7.5 Hz, 2H, H-3ʹ, H-5ʹ), 7.35 (d,* J* = 8.0 Hz, 2H, H-2ʹʹʹ, H-6ʹʹʹ), 7.33 (s, ^1^H, H-6), 7.07 (d*, J* = 8.0 Hz, 2H, H-3ʹʹʹ, H-5ʹʹʹ), 6.94 (s, ^1^H, H-3), 4.66 (br s, 2H, CH_2_-7ʹʹʹ), 3.59 (q, *J *= 7.0 Hz, 2H, CH_2_-1ʹʹ), 0.97 (t, *J *= 7.0 Hz, 3H, CH_3_-2ʹʹ).

*N-(3-Chlorobenzyl-N-(4,5-dibromo-2-ethoxyphenyl)benzenesulfonamide *(6i)

Brown amorphous powder, Yield 80%, m.p. 128-130 ^°^C,C_21_H_18_Br_2_ClNO_3_S,Mol. Mass-559.5; ^1^H-NMR (500 MHz, CDCl_3_): δ 7.69 (br d, *J* = 7.5 Hz, 2H, H-2ʹ, H-6ʹ), 7.58 (br t, *J *= 7.0 Hz, ^1^H, H-4ʹ), 7.47 (br t, *J *= 7.5 Hz, 2H, H-3ʹ, H-5ʹ), 7.35 (s, 1H, H-6), 7.19-7.18 (m, 3H, H-4ʹʹʹ, H-5ʹʹʹ, H-6ʹʹʹ), 7.10 (m, 1H, H-2ʹʹʹ), 6.95 (s, 1H, H-3), 4.68 (br s, 2H, CH_2_-7ʹʹʹ), 3.61 (br s, 2H, CH_2_-1ʹʹ), 1.0 (t*, J* = 6.5 Hz, 3H, CH_3_-2ʹʹ).

*N-(4-Fluorobenzyl-N-(4,5-dibromo-2-ethoxyphenyl)benzenesulfonamide *(6j)

Camel brown powder, Yield 85%, m.p. 150-152 ^°^C,C_21_H_18_Br_2_FNO_3_S,Mol. Mass-543; ^1^H-NMR (500 MHz, CDCl_3_): δ 7.69 (br d, *J* = 7.5 Hz, 2H, H-2ʹ, H-6ʹ), 7.57 (br t, *J *= 7.0 Hz, 1H, H-4ʹ), 7.46 (br t, *J *= 7.0 Hz, 2H, H-3ʹ, H-5ʹ), 7.30 (s, ^1^H, H-6), 7.15 (br d,* J* = 8.0 Hz, 2H, H-3ʹʹʹ, H-5ʹʹʹ), 6.94 (s, ^1^H, H-3), 6.90 (br d, *J* = 8.0 Hz, 2H, H-2ʹʹʹ, H-6ʹʹʹ), 4.68 (br s, 2H, CH_2_-7ʹʹʹ), 3.59 (br q, *J* = 7.0 Hz, 2H, CH_2_-1ʹʹ), 0.98 (t, *J* = 7.0 Hz, 3H, CH_3_-2ʹʹ).

*N-(2-Chlorobenzyl-N-(4,5-dibromo-2-ethoxyphenyl)benzenesulfonamide *(6k)

Brown granular solid, Yield 72%, m.p. 172-174^ °^C,C_21_H_18_Br_2_ClNO_3_S,Mol. Mass-559.5;^ 1^H-NMR (500 MHz, CDCl_3_): δ 7.70 (br d, *J* = 7.5 Hz, 2H, H-2ʹ, H-6ʹ), 7.57 (br t, *J *= 7.5 Hz, 1H, H-4ʹ), 7.53 (br d, *J* = 7.5 Hz, 1H, H-3ʹʹʹ), 7.47 (br t, *J *= 7.5 Hz, 2H, H-3ʹ, H-5ʹ), 7.43 (s, 1H, H-6), 7.24 (m, 1H, H-5ʹʹʹ), 7.20 (m, 1H, H-4ʹʹʹ), 7.15 (m,* J* = 7.3 Hz, 1H, H-6ʹʹʹ), 6.93 (s, 1H, H-3), 4.89 (s, 2H, CH_2_-7ʹʹʹ), 3.59 (br q, *J* = 7.0 Hz, 2H, CH_2_-1ʹʹ), 1.0 (t, *J* = 7.0 Hz, 3H, CH_3_-2ʹʹ).

*N-(4-Chlorobenzyl-N-(4,5-dibromo-2-ethoxyphenyl)benzenesulfonamide *(6l)

Brown amorphous powder, Yield 81%, m.p. 158-160 ^0^C,C_21_H_18_Br_2_ClNO_3_S,Mol. Mass-559.5; ^1^H-NMR (500 MHz, CDCl_3_) δ 7.68 (br d, *J* = 7.5 Hz, 2H, H-2ʹ, H-6ʹ), 7.57 (br t, *J *= 7.5 Hz, ^1^H, H-4ʹ), 7.46 (br t, *J *= 7.5 Hz, 2H, H-3ʹ, H-5ʹ), 7.33 (s, 1H, H-6), 7.20 (br d,* J* = 7.5 Hz, 2H,H-3ʹʹʹ,H-5ʹʹʹ), 7.12 (br d, *J* = 8.0 Hz, 2H, H-2ʹʹʹ, H-6ʹʹʹ), 6.94 (s, ^1^H, H-3), 4.68 (br s, 2H, CH_2_-7ʹʹʹ), 3.59 (br q, *J* = 7.0 Hz, 2H, CH_2_-1ʹʹ), 0.97 (t, *J* = 7.0 Hz, 3H, CH_3_-2ʹʹ).

*Biological activity*

*Acetylcholinesterase assay*

The AChE inhibition activity was performed according to the reported method (14) with slight modifications. Total volume of the reaction mixture was 100 μL. It contained 60 μL Na_2_HPO_4_ buffer with concentration of 50 mM and pH 7.7. Ten μL test compound (0.5 mM well^-1^) was added, followed by the addition of 10 μL (0.005 unit well^-1^) enzyme. The contents were mixed and pre-read at 405 nm. Then contents were pre-incubated for 10 min at 37 ^°^C. The reaction was initiated by the addition of 10 μL of 0.5 mM well^-1 ^substrate (acetylthiocholine iodide), followed by the addition of 10 μL DTNB (0.5 mM well^-1^). After 15 min of incubation at 37 ^0^C, absorbance was measured at 405 nm. Synergy HT (BioTek, USA) 96-well plate reader was used in all experiments. All experiments were carried out with their respective controls in triplicate. Eserine (0.5 mM well^-1^) was used as a positive control. The percent inhibition was calculated by the help of following equation )^19^ (.

$\mathrm{Inhibition}\left(\%\right)=\frac{\mathrm{Control}-\mathrm{Test}}{\mathrm{Control}}\times 100$

*Butyrylcholinesterse assay*

The BChE inhibition activity was performed according to the reported method (14,17) with slight modifications. Total volume of the reaction mixture was 100 μL containing 60 μL, Na_2_HPO_4 _buffer, 50 mM and pH 7.7. Ten μL test compound 0.5 mM well^-1^, followed by the addition of 10 μL (0. 5 unit well^-1^) BChE. The contents were mixed and pre-read at 405 nm. Then contents were pre-incubated for 10 min at 37 ^°^C. The reaction was initiated by the addition of 10 μL of 0.5 mM well^-1 ^substrate (butyrylthiocholine bromide), followed by the addition of 10 μL DTNB (0.5 mM well^-1^). After 15 min of incubation at 37 ^°^C, absorbance was measured at 405 nm. Synergy HT (BioTek, USA) 96-well plate reader was used in all experiments. All experiments were carried out with their respective controls in triplicate. Eserine (0.5 mM well^-1^) was used as a positive control. The percent inhibition was calculated by the help of following equation.

$\mathrm{Inhibition}\left(\%\right)=\frac{\mathrm{Control}-\mathrm{Test}}{\mathrm{Control}}\times 100$

IC_50 _values (concentration at which there is 50% enzyme inhibition) of compounds were calculated using EZ-Fit Enzyme kinetics software (Perella Scientific Inc. Amherst, USA). 

*Lipoxygenase assay*

Lipoxygenase activity was assayed according to the reported method (18-20) but with slight modifications. A total volume of 200 μL assay mixture contained 150 μL sodium phosphate buffer (100 mM, pH 8.0), 10 μL test compound and 15 μL purified lipoxygenase enzyme (Sigma, USA). The contents were mixed and pre-read at 234 nm and pre-incubated for 10 min at 25 ^°^C. The reaction was initiated by the addition of 25 μL substrate solution. The change in absorbance was observed after 6 min at 234 nm. Synergy HT (BioTek, USA) 96-well plate reader was used in all experiments. All reactions were performed in triplicates. The positive and negative controls were included in the assay. Baicalein (0.5 mM well^-1^) was used as a positive control. The percentage inhibition was calculated by formula as follows.

## Results and Discussion

*Chemistry*

**Scheme 1:**
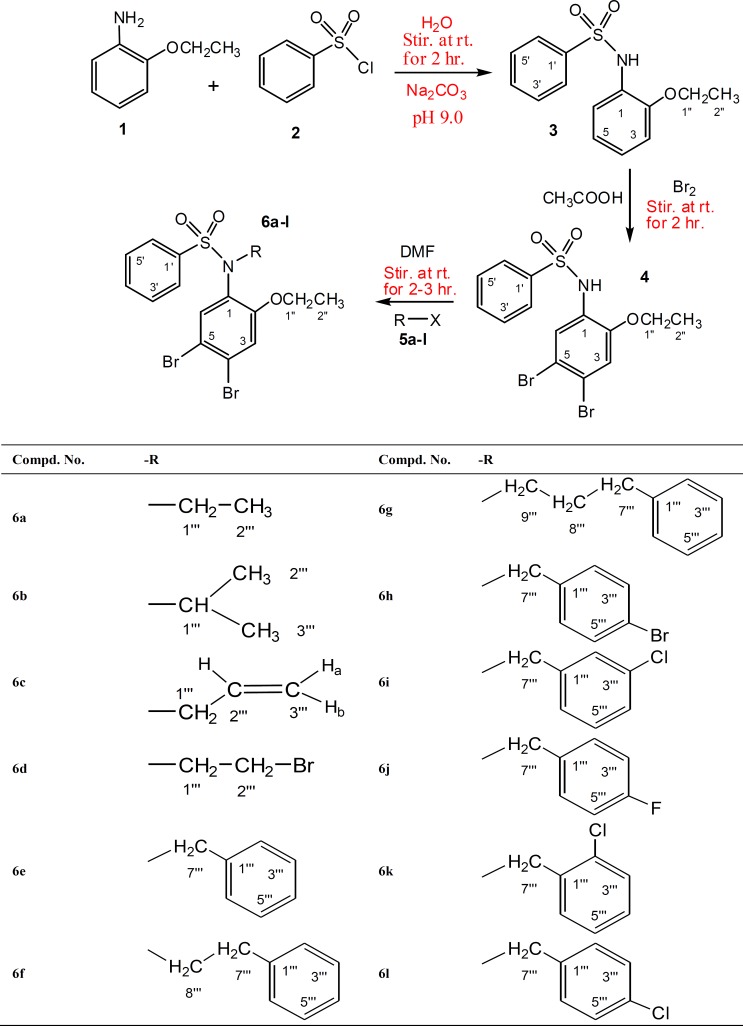
Synthesis of dibromosulfonamides 6a-l

The designed *N*-substituted new derivatives of brominated 2-phenitidine were synthesized according to scheme 1. First, a parent *i.e. N-*(4,5-dibromo-2-ethoxyphenyl) benzenesulfonamide (4) was prepared by bromination of *N*-(2-ethoxyphenyl) benzenesulfonamide (3) in glacial acetic acid at room temperature. The reaction was accomplished by stirring which gave the desired product in excellent yield. Second, the parent product 4 was further processed to achieve a series of new *N-*alkyl/aryl substituted sulfonamides 6a-6l. The brominated sulfonamide 4 was obtanined as a dark brown amorphous powder. The molecular formula C_14_H_13_NO_3_SBr_2_ was established by counting number of protons in ^1^H-NMR spectrum. Due to dibromination (21) of 2-phenitidine ring in this molecule, two singlets having integration of one proton each, appeared in its PMR spectrum at δ 7.77 and 6.91 which were assigned to the protons H-6 and H-3 respectively. The other signals in aromatic region appearing at δ 7.74 (dd, *J* = 1.5, 8.5 Hz, 2H, H-2ʹ, H-6ʹ), 7.53 (br t, *J* = 7.0 Hz, ^1^H, H-4ʹ), 7.43 (br t, *J* = 8.0 Hz, 2H, H-3ʹ, H- 5ʹ) were typical for the protons of the monosubstituted ring derived from benzenesulfonyl chloride. Furthermore, in aliphatic region a quartet at δ 3.78 (q, *J = *7.0 Hz, 2H, CH_2_-1ʹʹ) and a triplet at δ 1.24 (t, *J *= 7.0 Hz, 3H, CH_3_-2ʺ) were meticulous for an ethoxy group of substituted 2-phenitidine ring. On the basis of above cumulative evidences the structure of 4 was assigned as *N- *(4,5-dibromo-2-ethoxyphenyl)benzenesulfonamide which is a new synthetic sulfonamide. Similarly on the basis of spectral evidences from ^1^H-NMR, the structures of other derivatives of brominated 2-phenitidine were elucidated as described in experimental section and outlined in scheme-1.

*Biological activity*

The screening of the synthesized derivatives, 6a-6l, against acetylcholinesterase (AChE), butyrylcholinesterase (BChE) and lipoxygenase (LOX) enzyme demonstrated that all were active against butyrylcholinesterase and these also showed moderate inhibitory potential against acetylcholinesterase except 6a. Some of them also exhibited moderate inhibition against lipoxygenase (LOX) as it was evident from their IC_50 _values given in Table 1. 

**Table 1:** Enzyme inhibition studies of brominated 2- phenitidine derivatives (n = 3, mean ± sem).

**Compound** **No.**	**Acetylcholinestrase** (AChE)		**Butyrylcholinesterase** (BChE)		**Lipoxygenase** (LOX)	
	(%) at 0.5 mM	(IC_50_) μmoles/L	(%) at 0.5 mM	(IC_50_) μmoles/L	(%) at 0.5 mM	(IC_50_) μmoles/L
**4**	44.15±0.19	NIL	80.75±0.17	70.21±0.18	11.63±0.27	NIL
**6a**	28.87±0.31	NIL	85.33±0.26	54.91±0.85	81.75±0.71	152.91±0.22
**6b**	79.07±0.38	87.31±0.11	56.57±0.55	˃ 300	83.54±0.33	147.61±0.14
**6c**	72.22±0.55	143.91±0.08	80.75±0.68	68.41±0.21	66.26±0.54	226.71±0.21
**6d**	73.21±0.71	131.91±0.16	87.21±0.96	45.31±0.17	83.95±0.22	66.21±0.52
**6e**	79.27±0.63	88.21±0.17	85.92±0.28	58.71±0.08	31.29±0.18	NIL
**6f**	77.58±0.66	86.41±0.51	88.73±0.14	53.41±0.51	90.02±0.37	64.41±0.18
**6g**	77.18±0.33	158.41±0.11	90.14±0.77	57.51±0.63	75.21±0.11	132.91±0.17
**6h**	74.31±0.11	145.61±0.21	87.91±0.25	60.11±0.34	37.04±0.28	NIL
**6i**	82.54±0.36	73.91±0.87	91.78±0.33	57.11±0.46	49.59±0.64	NIL
**6j**	81.45±0.12	71.31±0.09	87.21±0.31	60.21±0.66	51.23±0.31	>400
**6k**	86.71±0.22	52.41±0.27	93.66±0.82	42.21±0.25	33.23±0.22	NIL
**6l**	82.94±0.15	126.61±0.02	66.31±0.11	190.31±0.17	55.45±0.84	> 400
**Control**	Eserine	0.04±0.001	Eserine	0.85±0.0001	Baicalein	22.4±1.3

Among these molecules *N-(2-Chlorobenzyl-N-(4,5-dibromo-2-ethoxyphenyl)benzenesulfonamide *(6k) and *N-(2-Bromoethyl-N-(4,5-dibromo-2-ethoxyphenyl)benzenesulfonamide *(6d) were found to be good inhibitors against butyrylcholinesterase (BChE) having IC_50 _value of 42.21 ± 0.25 and 45.31 ± 0.17 μmoles/L respectively, relative to eserine , a reference standard with IC_50 _value of 0.85 ± 0.0001 μmoles, probably due to substitution of 2-Chlorobenzyl and 2-Bromoethyl group, respectively in these compounds. Against acetylcholinestese, (6k) was too a good inhibitor comparative to others, having IC_50 _value of 52.41 ± 0.27 μmoles/L, relative to a reference standard eserine with IC_50 _value of 0.04 ± 0.001 μmoles/L, again presumebly due to substitution of 2-Chlorobenzyl moiety at *N*-atom in this molecule. However, the substitution of 2-Phenylethyl and 2-bromoethyl group, respectively, in *N-(2-Phenylethyl)-N-(4,5-dibromo-2-ethoxyphenyl) benzenesulfonamide *(6f) and *N-(2-bromoethyl)-N-(4,5-dibromo-2-ethoxyphenyl) benzenesulfon-amide *(6d) attributed these molecules as good inhibitors of lipoxygenase comparative to other derivates which was obvious from their lower IC_50 _values of 64.41 ± 0.18 and 66.21 ± 0.52 μmoles/L respectively, relative to baicalein, with IC_50 _value of 22.4 ± 1.3 μmolesL. The present investigation generally concluded that the synthesized brominated 2-phenitidine derivatives, 6a**-**6l, could be used as moderate inhibitors of cholinesterases and these are also ideally suited for further structural modification to obtain more potent and less cytotoxic therapeutic agents for the treatment of Alzheimer’s disease.
